# Therapeutics of Aconite and Iodine in Pericementitis

**Published:** 1894-04

**Authors:** W. I. Jones

**Affiliations:** Columbus


### Therapeutics of Aconite and Iodine in Pericementitis.

BY W. I. JONES, D.D.S., COLUMBUS.

Read before the Ohio State Dental Society, December, 1893.

Excepting toothache resulting from irritation of the pulp, there is no other pathological condition of the teeth so common, and at the same time so distressing, as inflammation of the peridental membrane, and whether it is caused by mechanical violence, poisonous infection, or by some chemical irritant, it is, in either case, unless arrested, as far-reaching in its results and as difficult of treatment, consequently for the cure of this lesion, many remedies have been advised. Among the *drugs* used are aconite and iodine; the first is a sedative to the nervous system, and when applied locally checks inflammation by paralyzing the nerves of the part and reduces the force of the circulation. It has been called the "*Therapeutic Lancet*" and, Potter says, rightly used, is one of the most valuable drugs we possess. Iodine is alterative, stimulant and tonic, promotes absorption of the products of inflammation and has been used for the cure of pleuritic effusion, ascites, hydrocele, ovarian dropsy and chronic abscesses. It acts especially as an excitant of the glandular and absorbent systems. Thus we see that one arrests further development of inflammation while the other diffuses the fluids already accumulated. The ways of administering are two: by placing in simple contact with the mucous membrane, and by hypodermic injection into the tissues over the affected teeth. The enepidermic method is, no doubt, universally employed. But the hypodermic administration is probably not practiced as widely as pericementitis is experienced. And because it has proved so effective in my own experience, am persuaded that it possesses advantages that the enepidermic does not. The dose is from three to four drops of tincture of aconite and iodine in equal parts injected into the loose tissue near the upper border of the alveolus. To insure the best result the point of the needle must be quickly passed beneath the mucous membrane and the contents of the syringe delivered slowly so as not to rupture the tissues. When done accordingly the operation becomes a very simple one. You have only to rightly manipulate the syringe and the drug is exactly where it is needed; is quickly absorbed; does not cause vesication and affords almost immediate relief. Such happy effects I have not obtained by any other remedy, and it is thus, only when the aconite and iodine are hypodermically administered.

---

In discussion of the above, Dr. W. H. Sedgwick says:

The peridental membrane being so abundantly supplied with vitality is frequently subjected to inflammation, and I know of no disease presented to the dental practitioner that is so annoying and perplexing as the one under consideration.

The peridental membrane is an elastic, velvet-like cushion, entirely lining the alveolar cavity of each root of the tooth. It has just enough of elasticity to allow the tooth a slight movement in any direction, when it resumes its normal position as the pressure is removed. This peculiar membrane is supplied with blood vessels and nerves, as is the pulp of the tooth at the apical foramen just before entering the pulp canal, these vessels separate and a branch continues along the side of each root, or fang, of the tooth, supplying the pericemental membrane. By the aid of this membrane we are enabled to distinguish the touch of the tooth, and Prof. Black, says " this is the only organ of touch possessed by the tooth, and this is so delicate that the least pressure of the tongue conveys the sensation to the tooth." By means of this delicate membrane we are enabled to distinguish this disease (pericementitis) from pulpitis and other diseases of the tooth that come to our notice—percussion producing pain and flinching, which is not the case in pulpitis.

Inflammation of the peridental membrane is ushered in and distinguished by redness of the gums, heat, pain and tumefaction or swelling. In diseases of this membrane we are in a measure confined to local applications to the gums—after removing the cause of disturbance, and here let me state that all inflammations are caused by an irritant; in pericementitis the irritant may be a putrescent pulp—may be from a blow, or some irritant around the gingival border of the alveolus—salivary calculus, for instance; and I have seen considerable inflammation of the membrane by the overlapping of an amalgam filling—probably inserted by myself—impinging on the peridental membrane at the neck of the tooth. Now before any therapeutic treatment will avail much, the cause of the irritation must be removed.

The treatment most relied on for pericementitis has been counter-irritation and stimulating the absorbents. Aconite and iodine have been largely depended on for this purpose, and I have always used the aconite and chloroform as a local application, applied by means of a napkin, at frequent intervals, followed by painting the gums with iodine; the iodine seems to be more readily absorbed and carried to the place where most needed, when the chloroform and aconite is first used; aconite paralyzes the sensory nerves, arterial tension is lessened, the inflammatory process being thus held in check until resolution ensues. Iodine acts also as a counter-irritant and excites the absorbents, thus whipping up these little scavengers, enabling them to carry off any effete matter accumulating in any local part of the body and diffusing it through the system; it has been extensively used in inflammation of the peridental membrane and of the gum tissue.

But, it may be inquired, "how are these external remedies brought to bear upon deep-seated and internal inflammations?" I answer that the beneficent author of our existence formed us so that there exists an intimate relation, a close sympathy, between internal parts and the external surface immediately opposite or over them, and physicians have all along availed themselves of its advantages in treatment of internal or deep-seated diseases. This treatment, to be effective, may have to be repeated for several days to secure control of the conditions. In my own practice I have found, in addition to the local applications, great benefit in administering aconite in one to two drop doses every hour for six or eight hours; or in the use of gelseminum in five drop doses. These are great depressors of the heart's action and should not be administered by ignorant or careless practitioners.

Dr. Jones, in his paper, has directed the attention of the profession to a new and novel method of applying these remedies to the seat of disease in pericementitis. Never having tried the hypodermic injection of iodine and aconite, I cannot speak advisedly of the practical value of this manner of using these remedies; but, theoretically, it seems to me to be applying the medicine exactly where needed, as the lymphatics will carry the remedy immediately to the parts diseased. The only criticism I would make is the dose of aconite; three or four drops injected into the loose tissues is a large dose, especially for some constitutions, and would have to be carefully watched; the use of iodine, hypodermically, would necessitate a new needle for almost every application.

But Dr. Jones' method certainly has its merits, and I hope will be tried by other members of the profession and reported on for the general good. I would add that Dr. Jones, in applying these remedies, dilutes them.

